# Emotional Blunting in Patients With Major Depressive Disorder: A Brief Non-systematic Review of Current Research

**DOI:** 10.3389/fpsyt.2021.792960

**Published:** 2021-12-14

**Authors:** Hongzhe Ma, Min Cai, Huaning Wang

**Affiliations:** Department of Psychiatry, Xijing Hospital, The Fourth Military Medical University, Xi'an, China

**Keywords:** major depressive disorder, antidepressants, emotional blunting, 5-HT, treatment optimalization

## Abstract

Emotional blunting is frequently reported by patients with major depressive disorder (MDD) and has been identified as one of the most prominent side effects of antidepressants leading to medication discontinuation. However, antidepressant-induced emotional blunting remains largely unexplored—there lacks a clinical definition of this condition, and no agreeing conclusion has been reached regarding its etiology. Current research suggests that the onset of diminished emotional response may be related to antidepressant dose, with higher doses being more likely to induce emotional blunting. Consequently, most clinicians either reduce the dose or switch to another drug when treating this symptom. Overall, more comprehensive clinical assessments or interviews specifically designed to evaluate antidepressant-induced emotional blunting in MDD patients are in need to elucidate the neuropsychological mechanisms behind this increasingly prevalent symptom.

## Introduction

Depressive disorders have been estimated to be the third leading cause of years lived with disability for all ages worldwide, and the global prevalence of MDD has significantly increased over the last few decades (1). The WHO predicts that by the year 2030, MDD will rank second in the Global Burden of Disease study as measured by disability-adjusted life years (2). By definition, MDD is a highly recurrent, comorbid, and chronic psychiatric disorder (3, 4) characterized by symptoms like persistently depressed mood, loss of interest or pleasure (anhedonia), and feelings of worthlessness or guilt (5). Pharmacological treatments, psychological interventions, and neuromodulation interventions are used to manage MDD symptoms and have been proven effective in various past studies (6–8).

Most evidence-based clinical practice guidelines recommend antidepressants as the first treatment step for MDD (9, 10). From 2005 to 2008, antidepressants were the third most commonly prescribed drug in the U.S., and the rate of antidepressant use increased nearly 400% from 1988 to 2008 (11). It has been estimated that the response rate to antidepressant treatments ranges from 42 to 53% (12, 13). Despite the medications' well-established treatment efficacy (14, 15), their side effects have also been documented in accumulating studies. In one online study conducted among 180 patients who were long-term users of antidepressants, more than 70% of the participants reported experiencing at least one side effect while taking their medications (16). In another study, 38% of 700 patients reported experiencing one or more side effects while taking selective serotonin reuptake inhibitor (SSRI) antidepressants, a relatively newer and safer generation of antidepressants (17, 18). Common side effects mentioned in these survey studies include headache, nausea, weight gain, and sleepiness (17, 19).

Emotional blunting is also considered a potential side effect of antidepressants, in particular SSRI antidepressants, and has been reported in multiple case reports and clinical studies. It was estimated that about 40–60% of patients who suffered from MDD and were treated with either SSRIs or serotonin-noradrenaline reuptake inhibitors (SNRIs) had experienced some degrees of emotional blunting (20, 21). In a recent survey conducted among 896 participants with depression (49.9% unipolar depression and 50.1% bipolar depression), emotional blunting was identified as one of the most prominent side effects leading to medication discontinuation in more than a third of the respondents, alongside lethargy, shaking/trembling, and anxiety (22). For more than 20 years, psychologists and psychiatrists have generally accepted that emotional blunting is a relatively common side effect of antidepressants (23, 24), and many patients strongly attribute their emotional symptoms either in total or in part to their medication (25). However, some studies challenge this view by stating that emotional blunting is more than a simple side effect of antidepressants—it also behaves like a residual symptom of depression (20). The following paper will provide an overview of current research on emotional blunting in patients with MDD, including its definition and assessments, the possible neuropsychological mechanisms underlying this clinical phenomenon, and potential treatments for emotional blunting in patients with MDD who showed an inadequate response to SSRIs/SNRIs.

## Definition

Emotional blunting is a condition present in many psychiatric disorders, including depression, schizophrenia (26) and post-traumatic stress disorder (27). As its name suggests, emotional blunting refers to a sense of numbing of both positive and negative emotions. It thus differs from anhedonia in a way that the latter refers to a markedly diminished interest or pleasure in activities, according to the Diagnostic and Statistical Manual of Mental Disorders, Fifth Edition (DSM-5) (5). Patients who suffer from emotional blunting are subject to a reduction in a broader range of emotions, including love, affection, fear, and anger (25). At the same time, emotional blunting also differs from apathy, a syndrome characterized by an absence of motivation that is not attributed to cognitive impairment, diminished level of consciousness, or emotional distress (28). In addition, apathy is increasingly recognized as a behavioral problem instead of an emotional side effect of a mental disorder (29, 30). Hence, diminished emotional responses associated with antidepressant treatment in patients with depression is a unique symptom and has been described as emotional blunting, emotional indifference, a reduction in emotional sensitivity, and a diminution in emotional responsiveness (24, 25, 29).

Many patients diagnosed with MDD and treated with SSRIs described a restriction or diminution in the intensity or frequency of emotions common in everyday living and essential for daily functioning (24, 25). For example, some people may become unable to cry, share others' sadness and joy, or even enjoy what they used to like. In a few extreme cases, patients described that they were unable to feel any emotions at all. Others felt that their emotions had become more “cognitive” and more like their thoughts rather than feelings. In this way, emotional blunting may pose a risk to the adherence to antidepressant treatments and lead to more serious outcomes, like reduced quality of life or reduced social responsibilities (22).

## Psychometric Assessments

Despite lacking a clinically preferred scale and a clear clinical definition for antidepressant-induced emotional blunting (29), a few questionnaires have been developed to diagnose, assess, and quantify the severity of this specific condition. The Laukes Emotional Intensity Scale (LEIS) is one of the first instruments to successfully measure SSRI-induced emotional blunting (38). It is a self-report instrument aiming to assess the intensity of emotions, regardless of the frequency with which these emotions are experienced. The entire assessment consists of 18 questions rated in relation to the respondent's “usual” state on a 5-point scale (1 = a lot less, 2 = somewhat less, 3 = same as usual, 4 = somewhat more, and 5 = a lot more). In a past study investigating emotional blunting and SSRI-induced sexual dysfunction, the LEIS was used to measure SSRI-induced emotional blunting (24). Results showed significant reductions in 12 out of 18 emotions, with the most prominent reductions in sexual interest, pleasure, and erotic dreams. However, the instrument was not used in any later studies at the time of this review.

A more recently developed instrument is the Oxford Questionnaire on the Emotional Side-effects of Antidepressants [OQuESA or OQESA; (33)]. It is a self-report questionnaire comprising three sections for a total of 26 items focusing on the patient's emotional experience over the past week. Section one has 12 items and explores the respondent's current experience of emotional blunting. Section two consists of eight items aiming to relate the respondents' current emotional states with their normal emotional states prior to depression. Lastly, section three a Residual Symptom of MDD comprises six items asking the patients to what extent they believe there is an association between their current antidepressant medication and emotional blunting and whether this affects treatment compliance. Each item is rated on a 5-point scale ranging from 1 (disagree) to 5 (agree). The OQuESA was tested on a previous sample of depressed patients and demonstrated high construct validity and reliability. Later studies employing this instrument all showed significant differences in scores between patients with and without emotional blunting (20, 39).

One limitation with all current psychometric assessments on antidepressant-induced emotional blunting is that there is no clinical definition or a standard measure of emotional blunting, making it difficult to test the accuracy of these evaluations (33). Moreover, the instruments are all self-reported and rely heavily on the patients' interpretations. Thus, questionnaires administered by trained professionals should also be developed to ensure the respondents have interpreted the questions correctly.

## A Residual Symptom of MDD?

A substantial amount of subjective evidence suggests that emotional blunting is induced by antidepressants and is different from the emotional states experienced during depressive episodes (23, 31). For example, in one qualitative study, experimenters interviewed 220 participants who had been depressed and were either taking or had taken SSRIs (25). Most respondents specifically attributed the numbing of emotions to their medication. However, several patients were uncertain whether it was the SSRI they took or changes in life events that had caused their emotional blunting.

Some researchers argue that emotional blunting is not only a simple side effect of antidepressants but also a residual symptom of depression. They believe that emotional blunting is a symptom of depression that is not measured in conventional scales and is often incompletely treated with SSRIs (32). In one experiment, 669 patients with depression who were taking antidepressants at the time were recruited (20). A group of 150 recovered controls was also included in the analysis. All subjects were asked to complete the Hospital Anxiety and Depression (HAD) scale, as well as the OQuESA to assess emotional blunting. The HAD-D subscale measures the severity of current depressive symptoms, and the results showed that higher HAD-D scores correlated with the presence of emotional blunting, indicating it might be a residual symptom of depression rather than simply a side effect of antidepressant treatment. The researchers proposed that to determine whether emotional blunting is indeed a residual symptom of depression, future studies could evaluate the presence of emotional blunting in healthy subjects taking antidepressants (20, 33). The absence of blunted feelings would confirm emotional blunting is not induced by antidepressants.

A later study was conducted among 17 healthy volunteers who received either 7 days of bupropion, a norepinephrine-dopamine reuptake inhibitor, or placebo (34). Contrary to the group's previous study on citalopram (SSRI) and reboxetine (SNRI), in this study, the bupropion group showed increased resting-state functional connectivity (RSFC) in the dorsal medial prefrontal cortex (dmPFC) areas instead of diminished RSFC (35, 36). The dmPFC is thought to be involved in emotional processes, particularly the intensity of emotions (37). Together, these results provide a potential explanation for the lower rate of emotional blunting (33%) in patients taking bupropion (20) and suggest that antidepressants can indeed induce changes in neural mechanisms underlying diminished emotional experiences, even in healthy controls without depression.

Nevertheless, emotional blunting is a relatively subjective symptom, and studies directly measuring emotional blunting in the form of interviews or questionnaires should also be conducted to determine whether the symptom occurs in healthy controls taking antidepressants. In all, although most studies agree that antidepressant treatment induces emotional blunting in patients with MDD, a few studies suggest that the numbing of emotions could also be a residual symptom of MDD due to incomplete treatment.

## Neuropsychopharmacological Mechanisms

The first antidepressants were discovered by chance more than 60 years ago and were later found to promote serotonin (5-HT) or noradrenaline (NA) function in the central nervous system (40–43). Almost all later discovered antidepressants were developed to act on 5-HT and NA neurotransmitters, with only a few exceptions (44, 45). Despite antidepressants' relatively well-studied mechanisms of action, no agreeing conclusion has been reached regarding the precise etiology of antidepressant-induced emotional blunting. A number of possible explanations have been proposed, and the majority of them focus on SSRI-induced emotional blunting. For example, in an early case report, the authors documented the occurrence of apathy and indifference in a patient taking high doses of fluoxetine (46) and noted a decreased cerebral blood flow in the frontal lobes and changes in neuropsychological tests intended to measure frontal lobe dysfunctions. Two theories have gained increasing attention among all explanations for the onset of antidepressant-induced emotional blunting (29).

One explanation suggests that SSRIs may alter frontal lobe activity *via* serotoninergic effects (47). SSRIs increase 5-HT levels in the brain by inhibiting the reuptake of 5-HT (48). Of all cortical regions, the frontal lobe, which is highly related to human emotional responses, exhibits the highest density of serotoninergic axons and 5-HT receptors (49, 50). The localization of 5-HT receptors in the frontal lobe thus indicates the critical role of 5-HT in emotional functions and mood regulation. Indeed, in a group of psychiatrically healthy subjects, experimenters noticed that serotoninergic functioning could exert inhibitory influence over both positive and negative emotions (51). Moreover, animal experiments demonstrate that endogenous release of 5-HT can modulate neural oscillations in the prefrontal cortex (PFC), further supporting the role 5-HT plays in controlling frontal lobe activity (52).

The other explanation proposes that SSRIs act on the serotoninergic systems, which in turn modulate midbrain dopamine (DA) systems that project to the prefrontal cortex (29, 53). As evident in past studies, DA neuronal cell bodies and axon terminals are modulated by 5-HT and receive extensive projections from these neurons (54). Consequently, by altering 5-HT levels in the brain, DA activities will also be affected. Furthermore, midbrain DA neurons constitute the mesocorticolimbic pathway critical for creating rewards and signaling aversive outcomes (55, 56). Rewards are able to elicit pleasurable feelings, while aversive stimuli induce negative emotions (57). Alterations in DA levels can thus explain the numbing of both positive and negative emotions. This theory is further supported by a clinical study, where participants receiving citalopram (SSRI) showed reduced prefrontal cortex activation to both rewarding and aversive stimuli, while participants receiving placebo and reboxetine (SNRI) did not demonstrate suppressed responses (35). The results are consistent with previous findings showing a mutually opposed relationship between 5-HT and DA systems (58). In this way, alterations in DA levels due to increased 5-HT release exert inhibitory effects on rewarding and aversive stimuli, which could explain the subjective experience of emotional blunting in some patients taking SSRIs.

As mentioned earlier, almost all current explanations for antidepressant-induced emotional blunting in patients with MDD revolve around SSRIs and the neurotransmitter serotonin. However, patients taking other types of antidepressants have also reported blunted emotions as a side effect of antidepressant use (20). Thus, future work elucidating the neural mechanisms underlying the onset of emotional blunting in patients taking other classes of antidepressant is in need.

## Clinical Management and Treatment

On the one hand, antidepressants successfully reduced the emotional distress patients experienced during their depressive episodes; on the other hand, unwanted outcomes like reduced sociability and emotional detachment from family urge managements targeting the emotional side effect of antidepressants. The first option to consider is reducing the dosage or discontinuing the medication (29, 53). Some patients noticed reduced emotional responses while on their SSRIs and returned to their normal emotional states after discontinuing the medication or lowering the dose (25). As mentioned in a case report study, one patient with depression received fluoxetine as her medication (23). Within 7 days, she noticed being emotionally blunted and complained about a sense of apathy. To resolve this issue, she continued on fluoxetine due to significantly improved mood but slowly lowered the dose. Eventually, she noticed a partial improvement in her symptoms when taking fluoxetine 2omg 4 days per week. Overall, the occurrence of emotion blunting seems to be dose-related, with lower doses being less likely to induce this symptom.

The second strategy is switching to a medication in a different drug class (29, 53). In the same case report study mentioned above, two patients received fluoxetine as their medication and complained about a sense of apathy due to the drug (23). Because of these new emotional experiences, they eventually discontinued fluoxetine and started either on tranylcypromine, a monoamine oxidase inhibitor, or amitriptyline, a tricyclic antidepressant. As a result, symptoms disappeared within several weeks. In more recent studies, emotional blunting has been found to be less frequent in patients taking agomelatine than escitalopram (SSRI) and less severe when medication was switched from SSRIs/SNRIs to vortioxetine (32, 39). Unlike traditional SSRIs and SNRIs, agomelatine is a melatonin agonist and 5HT_2c_ antagonist, while vortioxetine is often classified as a serotonin modulator and stimulator (59, 60). The multimodal antidepressant vortioxetine has also shown significant effects on alleviating anhedonia symptoms in patients with MDD (61). Although, anhedonia is identified as a different class of symptoms, the phenotypical overlap between anhedonia and emotional blunting indicates that their treatment strategy may be similar (39). Overall, findings from both emotional blunting and anhedonia studies suggest that switching from SSRI/SNRIs to other monoaminergic agents or multimodal antidepressants could be a promising maneuverer for managing emotional blunting (62, 63).

Augmentation, or the addition of a second drug, has also been suggested as a treatment strategy (29, 53). In one case report, a patient diagnosed with mild MDD was initially treated with fluoxetine (64). Over the next month, she appeared flat in emotions and complained about being “mentally tired.” Her treating psychiatrist lowered the dose of fluoxetine and added bupropion to her medication, which normalized her affect in 2 months. However, it is not clear whether the improvement was due to a reduction in fluoxetine dose or augmentation with bupropion. Thus, the first two strategies are more commonly adopted by clinicians.

Lastly, neuromodulation interventions may also be effective in improving the symptoms of antidepressant-induced emotional blunting. Although the precious etiology of this clinical phenomenon remains unclear and evidence from neurostimulation studies is lacking, it is generally accepted that the prefrontal cortex plays a critical role in regulating emotions (65). Furthermore, past studies investigating the effects of non-invasive brain stimulations on anhedonia demonstrated that by stimulating the prefrontal cortex, improvements in anhedonia symptoms could be observed (66–68). Nonetheless, anhedonia and emotional blunting are not the same, and future research is needed to determine whether neuromodulation is a potential treat option for antidepressant-induced emotional blunting.

## Conclusion

Emotional blunting has been reported by many patients diagnosed with MDD, but its neuropsychological basis remains indefinite. Most patients specifically attribute this symptom to their antidepressant medication; however, some psychologists believe that emotional blunting is also a residual symptom of depression due to incomplete treatment. Despite lacking a standard definition for antidepressant-induced emotional blunting, questionnaires like The Laukes Emotional Intensity Scale and the Oxford Questionnaire on the Emotional Side-effects of Antidepressants have been used in current studies assessing the intensity and severity of emotional blunting due to antidepressant use. As for treatment options, both reducing the antidepressant dose and changing to a medication in a different drug class are used by clinicians to alleviate the symptom. Overall, emotional blunting in patients with MDD remains largely unexplored. The high prevalence of this symptom calls for a standard clinical definition and more comprehensive and targeted clinical assessments, and future studies elucidating the mechanisms that underlie emotional blunting are in need.

## Author Contributions

All authors listed have made a substantial, direct, and intellectual contribution to the work and approved it for publication.

## Funding

This work was supported by grants from the National Natural Science Foundation of China (Nos. 81701145 to MC and 81974215 to HW).

## Conflict of Interest

The authors declare that the research was conducted in the absence of any commercial or financial relationships that could be construed as a potential conflict of interest.

## Publisher's Note

All claims expressed in this article are solely those of the authors and do not necessarily represent those of their affiliated organizations, or those of the publisher, the editors and the reviewers. Any product that may be evaluated in this article, or claim that may be made by its manufacturer, is not guaranteed or endorsed by the publisher.
